# Hearing in *Drosophila*

**DOI:** 10.1016/j.conb.2015.02.001

**Published:** 2015-10

**Authors:** Jörg T Albert, Martin C Göpfert

**Affiliations:** 1 Ear Institute, University College London, 332 Gray's Inn Rd, London WC1X 8EE, UK; 2 Department of Cellular Neurobiology, University of Göttingen, Julia-Lermontowa-Weg 3, 37077 Göttingen, Germany

## Abstract

The dissection of the *Drosophila* auditory system has revealed multiple parallels between fly and vertebrate hearing. Recent studies have analyzed the operation of auditory sensory cells and the processing of sound in the fly's brain. Neuronal responses to sound have been characterized, and novel classes of auditory neurons have been defined; transient receptor potential (TRP) channels were implicated in auditory transduction, and genetic and environmental causes of auditory dysfunctions have been identified. This review discusses the implications of these recent advances on our understanding of how hearing happens in the fly.

## Highlights

• Dissecting sound transduction and auditory signal processing.
• Defining transcription factors that organize auditory cilia and auditory neuron wiring.
• Elucidating environmental and genetic causes of hearing impairments.
• Delineating the genetic repertoire of auditory sensory cells.

## Graphical abstract

**Figure:**
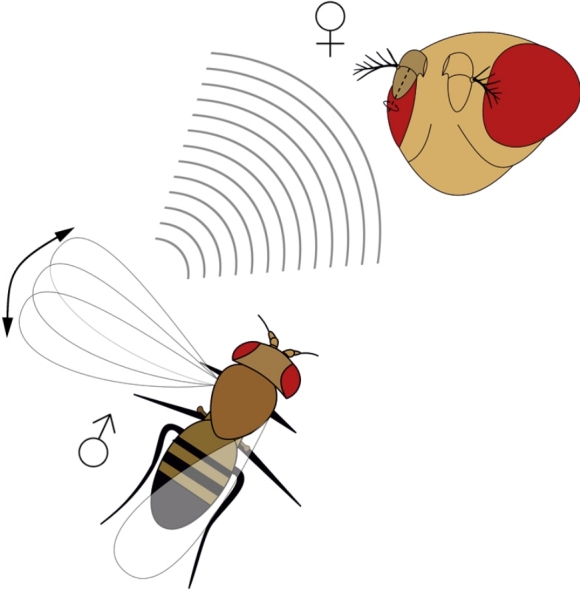

**Current Opinion in Neurobiology** 2015, **34**:79–85

This review comes from a themed issue on **Molecular biology of sensation**

Edited by **David Julius** and **John R Carlson**

For a complete overview see the Issue and the Editorial

Available online 22nd February 2015

**http://dx.doi.org/10.1016/j.conb.2015.02.001**

0959-4388/© 2015 The Authors. Published by Elsevier Ltd. This is an open access article under the CC BY license (http://creativecommons.org/licenses/by/4.0/).

## Introduction

**Figure 1:**
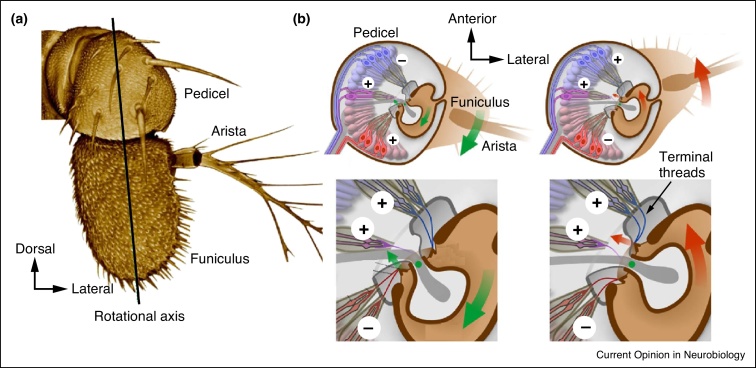
*Drosophila* hearing organ and direction sensitivities of JONs. **(a)** Frontal view of the *Drosophila* antenna. When acoustically stimulated, the arista and the funiculus sympathetically vibrate about the longitudinal axis, thereby activating JONs in the pedicel of the antenna. **(b)** Cross-sections through the pedicel-funiculus joint (top: overviews, bottom: zoom-ins), depicting the funicular connection sites of JONs. Deflecting the antenna posteriorly stretch-activates (depolarizes) deflection-sensitive JONs that connect to the posterior side of the funiculus (left) but inactivates (hyperpolarizes) JONs connecting to its anterior side. For posterior deflections of the antenna, the signs of activation are inversed. Auditory JONs might be equally activated by anterior and posterior antennal movements; judging from their somata positions, they might connect medially to the funiculus with their terminal threads.

Modified from Ref. [16].

**Figure 2:**
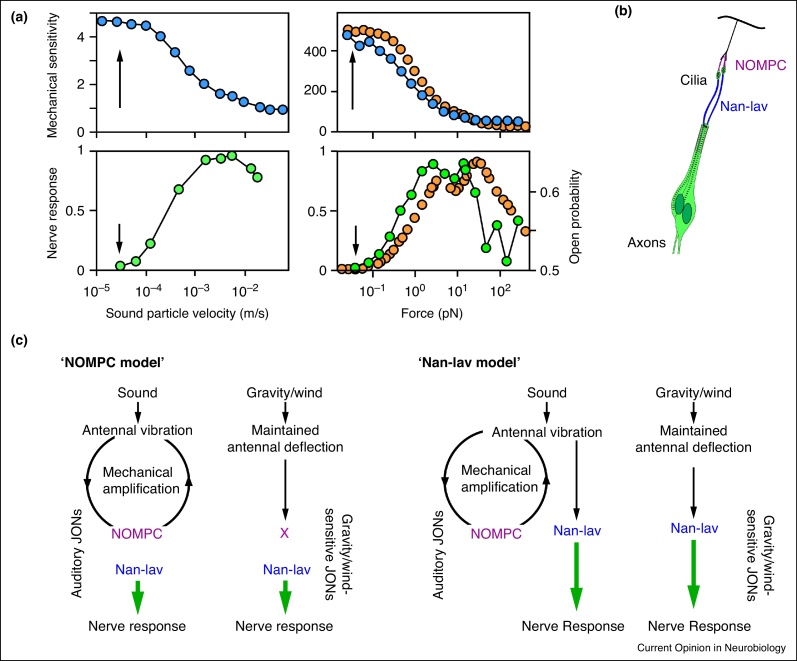
Transduction and amplification. **(a)** Left: mechanical sensitivity of the antenna (measured as antennal vibration velocity (m/s) normalized to the sound particle velocity (m/s)) as a function of the sound particle velocity (top), and corresponding relative amplitude of the sound-evoked antennal nerve potentials (bottom). Mechanical amplification by JONs maximally enhances the antenna's sensitivity to faint sounds (arrow, top) that, by themselves, would be too weak to evoke nerve potentials (arrow, bottom) (adopted from Ref. [15^••^]). Right: maximum sensitivity to faint sounds is also seen when the antenna's mechanical sensitivity is measured as the ratio between antennal displacement (nm) and the force (pN) that, during sound stimulation, is experienced by the antenna (Top). This mechanical behavior and also the amplitude characteristics of the nerve response (bottom) are reproduced by an active version of the gating spring model (orange circles) that links mechanical amplification by JONs to the open probability of MET channels (bottom) (adopted from Ref. [19]). **(b)** Localization of NOMPC and Nan–Iav in JON cilia (see also Refs. [28–30]). **(c)** Transduction models. According to the ‘NOMPC model’ (left), auditory JONs use NOMPC to transduce and mechanically amplify vibrations, and gravity/wind-sensitive JONs transduce antennal deflections with a second, unknown channel (‘X’). Downstream of transduction, electrical signals are amplified by Nan–Iav. The Nan–Iav model (bottom) posits that Nan–Iav mediates transduction in auditory and gravity/wind-sensitive JONs. NOMPC acts as a mechanical pre-amplifier in auditory JONs that, together with motor proteins, augments vibrations prior to transduction (see also Ref. [26]).

**Figure 3:**
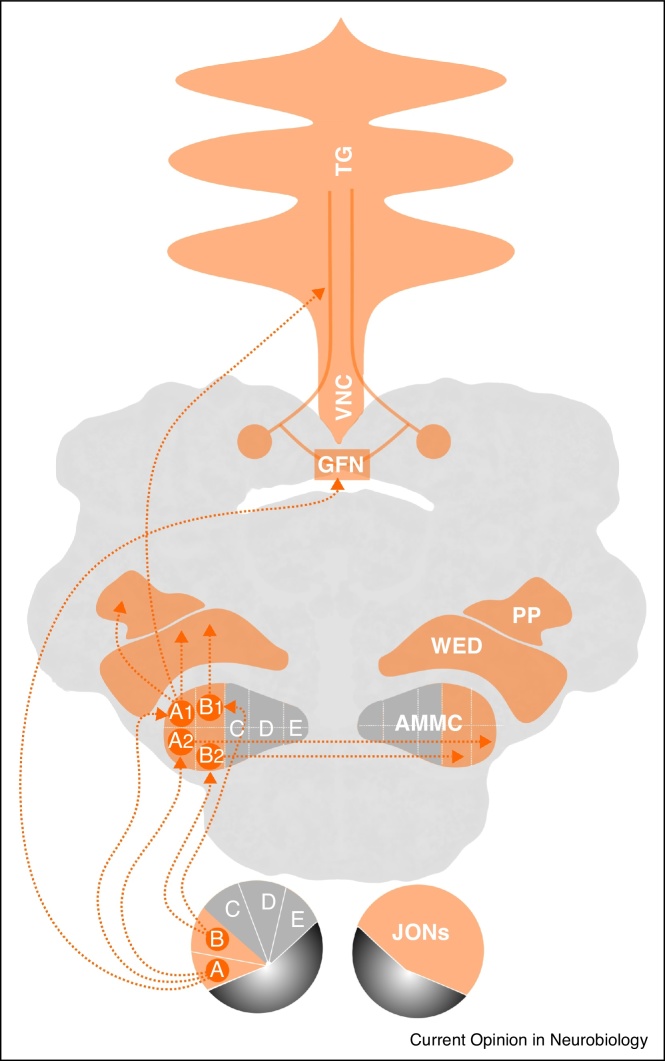
The main auditory circuit in *Drosophila*. Hearing starts with JONs, the first order auditory neurons in the pedicel of the antenna. Two classes of JONs, JON-A and JON-B, have been specifically linked to auditory transduction and sound-induced behavior. JON-A and JON-B target second-order auditory neurons in different zones of the antennal mechanosensory motor center (AMMC-A and AMMC-B, respectively), the ventral nerve cord (VNC) and the thoracic ganglia (TG); partly, they also make contact with the giant fiber network (GFN) via electric synapses (predominantly JON-A). The second-order auditory neurons in the AMMC either form auditory local neurons (aLNs), which arborize exclusively within the AMMC without projecting to other brain regions (omitted here for the sake of clarity) or auditory projection neurons (aPNs). Some aPNs (AMMC-A1 and AMMC-B1) target third-order auditory neurons in the wedge (WED) and the posterior protocerebrum (PP), others (AMMC-A2 and AMMC-B2) send commissural projections to the respective contralateral AMMC zone. Please note that, in the interest of clarity, projections from only one hemisphere are shown (left side of diagram). Based on Refs. [7,8,10,39,41,42,44^••^].

Hearing in *Drosophila melanogaster* serves the detection of the courtship songs male flies produce by fanning one of their wings [1]. These close-range songs, the spectral composition of which matches the flies’ range of hearing (ca. 100–300 Hz), drive female mating decisions [2] and stimulate other males to court and sing [1,3]. Both sexes detect sounds with Johnston's organ (JO) (Figure 1) — an array of ca. 500 chordotonal stretch-receptor neurons (JONs) in the pedicel of the fly's antenna [4,5] (Figure 1a). It is currently not known if there are any sexual dimorphisms in JO. Each JON bears a single ciliated dendrite, which transduces stimulus-induced antennal displacements into electrical currents [6], and an axon that propagates the resulting action potentials to the antennal mechanosensory motor center (AMMC) in the deuterocerebrum of the fly's brain [7^••^] (Figures 2b and 3). This review discusses recent advances in our understanding of JON function and the central auditory circuitry downstream of JONs.

## Mechanically evoked JON responses

On the basis of their axonal target regions in the AMMC, the fly's ca. 500 JONs can be categorized into five classes, labeled A–E [7^••^]. Whereas the ca. 200 JONs of classes A and B mainly respond to sound-induced antennal vibrations and are required for hearing, the ca. 250 JONs of classes C and E preferentially respond to maintained antennal deflections and serve the detection of gravity and wind [8^••^,9^••^]. By analyzing population calcium responses, Matsuo *et al.* [10] have recently reported that the ca. 50 class D JONs respond to both vibrations and deflections of the antenna; the neurobiological relevance of this dual response behavior, however, is still unclear. Recent reports implicating JONs in *Drosophila* flight control [11,12] might suggest that the flies use class D JONs to monitor both wind and wing-beat sounds generated during flight. Such proprioceptive role would explain why vibration and deflection amplitudes of several micrometers seem required to activate these neurons [10], whereas vibrations of 50 nm suffice to elicit responses of the auditory JONs of classes A and B [13]. Antennal vibrations exceeding 200 nm also activate the JONs of classes C and E, which, in addition to detecting wind and gravity, might contribute to hearing when sounds are intense [13].

JON classes also differ in their direction-sensitivities, reflecting their perpendicular connection to the opposing sides of the antenna (Figure 1b). Connecting to the antenna's posterior side, JONs of class E are stretch-activated when the antennal is deflected backwards, whereas forward deflections stretch-activate class C and D JONs that mostly seem to connect to the antenna's anterior side [8^••^,9^••^,10]. Both forward and backward movements of the antenna are expected to equally stretch-activate the auditory JONs of classes A and B, which — as judged by the positions of their somata — might connect medially to the antenna [14–16]. Testing for such bidirectional activation will require measurements of single cell responses and more detailed information about their antennal connection sites: these sites cannot be inferred from somata positions because JONs are tethered to the antenna by curved terminal threads [17] (Figure 1b).

Vibration-sensitive JON classes further differ in their frequency-characteristics [8^••^,9^••^,10], pointing to cell-intrinsic tuning mechanisms, in addition to the frequency filtering that is provided by the antenna's resonant mechanics. The latter antennal mechanics was shown to be actively modulated by motile responses of JONs, which match the antenna's resonance to the courtship song frequencies by actively augmenting the antennal vibrations in frequency-dependent and intensity-dependent ways [18–21]. Targeted cell ablations revealed that this active mechanical amplification requires the auditory class A and B neurons, but not the gravity/wind-sensitive JONs of classes C and E [13]. Testing whether all the auditory JONs exert mechanical amplification will require single cell approaches as there might be a certain degree of functional heterogeneity even within one class of JONs.

## Mechano-electrical transduction and amplification

Antennal displacements are coupled via the terminal threads to the mechanosensory cilia of JONs, where they gate mechano-electrical transduction (MET) channels [22]. This gating introduces a nonlinear compliance into the fly's antennal mechanics that, conforming to the gating spring model of vertebrate auditory transduction [23], suggests that the MET channels are directly gated by pull of gating springs. The interplay between this mechanogating and associated motor movements quantitatively explains mechanical amplification in *Drosophila* hearing [19], indicating that the same transducer-based mechanism that drives active hair bundle movements in vertebrate hair cells [24] also promotes the motility of JONs. The mechanistic link between transduction and amplification by JONs was recently put into question because ‘active amplification is observable for intensities below the threshold for antennal field potential responses’ [15^••^] (Figure 2a). Neither field potentials nor channel gating, however, possess thresholds, and a transduction-based model well captures the intensity-dependence of amplification in the fly's ear [19] (Figure 2a).

Recent studies have supported TRP channels as the candidate MET channels of JONs, and two transduction models were proposed [13,15^••^,25^••^,26,27] (Figure 2c): The ‘NOMPC model’ posits that the NOMPC (=TRPN1) channel mediates transduction in the auditory JONs, participating in mechanical amplification and mediating sensitive hearing [25^••^,26,27]. Gravity/wind-sensitive JONs are assumed to harbor a second, less sensitive MET channel whose mechanogating is independent of NOMPC. Downstream of the MET channels, electrical signals are amplified by the two TRPV channels Nan and Iav, which are required for electrical signaling by JONs and localize downstream of NOMPC in JON cilia (Figure 2b), presumably forming Nan–Iav heteromers [28–31]. The ‘Nan–Iav model’ [15^••^,26] posits that transduction is mediated by Nan–Iav. In this scenario, NOMPC acts as a mechanical pre-amplifier in auditory JONs that enhances auditory sensitivity by enhancing vibrations before they are transduced in auditory JONs.

Both models can explain why loud sounds still evoke residual antennal nerve potentials in *nompC* null mutants [13]. According to the ‘NOMPC model’, loss of NOMPC abolishes sensitive sound-transduction in auditory JONs, but louder sounds still activate the less sensitive MET channels in gravity/wind-sensitive JONs [22,26]. In the Nan–Iav model, transduction persists in both sound-sensitive and gravity/wind-sensitive JONs, and it is the loss of mechanical amplification that explains the drop in auditory sensitivity [15^••^].

NOMPC was recently established as a *bona fide* MET channel that can be gated directly by mechanical stimuli *in vitro* and confer cellular mechanosensitivity *in vivo* [32^••^,33^•^]. Nan and Iav can be reportedly activated by hypertonicity [28,29], indicating that they are mechanosensitive. Testing whether mechanically stimuli directly activate Nan and Iav will require further experimentation, and so does the activation mechanism of the putative Nan–Iav heteromers. If the ‘Nan–Iav model’ were correct, one would expect that mechanical stimuli directly activate Nan–Iav, whereas in line with the ‘NOMPC model’ one would expect Nan–Iav to be voltage-gated because signaling from NOMPC to Nan–Iav seems to too fast to allow for diffusible messengers [15^••^,26]. Apart from NOMPC and Nan–Iav, also other MET channels need to be considered, including for example the *Drosophila* Piezo channel that seems present in some JONs [34^••^].

## Organizing JON cilia and axonal wiring

The proper localization of NOMPC and Nan–Iav in JON cilia was recently found to require the Tubby-like protein (TULP) family member dTulp [35^•^], whose mouse homologue is implicated in cilium organization [36] and cochlear integrity [37]. Disrupting *Drosophila* dTulp abolished the ciliary localization of Nan–Iav and mislocalized NOMPC to the proximal ciliary region [35^•^]. Loss of Nan–Iav in JON cilia was also observed in mutants lacking the forkhead transcription factor Fd3F, which, together with the transcription factor RFX, organizes the expression of mechanosensory relevant ciliary proteins in the cilia of JONs [38^•^]. Another transcription factor, the homeodomain transcription factor Engrailed (EN), was found to be involved in the wiring of JONs to downstream neurons in the AMMC [39^•^]. EN is expressed in a subset of auditory JONs that form electrical synapses with the giant-fiber neuron (GFN). Misexpressing En in En-negative gravity/wind-sensitive JONs induced ectopic chemical and electrical synapses with the GFN, whereas RNAi-mediated knockdown of En in En-positive JONs reduced the strengths of their synaptic connections with the GFN. Intriguingly, EN organizes the patterning of retinal axon terminals in the vertebrate midbrain [40], similar to its role in auditory JONs.

## Central circuitries and sound processing

JONs of classes A–E target different zones in the AMMC where they synapse onto different second-order neurons (Figure 3). Five classes of interneurons were identified that receive input from the auditory JONs of classes A and B [8^••^,41,42^••^]: firstly, the GFN, which conveys auditory information to the thoracic ganglia and the inferior ventrolateral protocerebrum (IVLP; also referred to as *wedge* or WED [43]); secondly, the AMMC-A1 and thirdly, the AMMC-B1 neurons, which connect the JONs of classes A and B to the IVLP/WED, respectively; fourthly, the AMMC-A2 and lastly, AMMC-B2 neurons, which seem to connect the respective AMMC-zones between both hemispheres. A recent large-scale anatomical screen [44^••^] confirmed these projection patterns and reported distinct groups of candidate auditory projection neurons (aPNs), which either, firstly, arborize within AMMC zone A sending projections to the posterior protocerebrum (PP) or, secondly, arborize within AMMC zone A sending projections to the ventral nerve cord (VNC) or, thirdly, arborize within AMMC zones A and B sending projections to the VNC or, lastly, arborize within AMMC zone B sending bilateral projections to the IVLP/WED. The same screen also identified classes of candidate auditory local neurons (aLNs) that arborize within either, firstly, AMMC zone A, secondly, AMMC zone B or thirdly, AMMC zone A and B. Only two classes of interneurons, aPN1 (AMMC-B1 from Ref. [8^••^]) and aLN(al), both of which receive their dendritic inputs exclusively from AMMC zone B, were found to be necessary for behavioral responses to courtship songs in both females and males. Class D JONs, which might also contribute to hearing, were found to target AMMC-D1 neurons as well as local interneurons that are confined to the AMMC [10]. The AMMC-D1 neurons arborize within the AMMC and send projections into the thoracic ganglia [10].

Functional studies have begun to uncover the response characteristics of these second-order auditory interneurons [41,42^••^,44^••^]. Many of the neurons were reported to be non-spiking [41], and AMMC-B1 neurons, for example, could be classified into four subtypes that differ in their sensitivities and frequency characteristics [42^••^]. Progress has also been made with respect to next stage of neuronal sound processing. Within the VLP, first third-order auditory neurons were identified that connect the IVLP/WED to the posterior part of the VLP [42^••^]. This posterior part of the VLP displays glomerular structures and, receiving also visual and gustatory input, might integrate multimodal stimuli [42^••^].

## Auditory dysfunctions and novel proteins for hearing

Within the past years, multiple causes of JON dysfunctions have been identified, including genetic ones and acoustic noise. Evidence for noise-induced hearing loss has been reported by Christie *et al.* [45^••^], who exposed flies for one day to very intense tones at a frequency of 250 Hz. Immediately after exposure, sound-evoked nerve potentials displayed longer latencies and reduced amplitudes. Normal response latencies and amplitudes reappeared within one week, yet then JON mitochondria had become smaller, which is indicative of metabolic stress [45^••^]. Collectively, these noise effects are reminiscent of noise-induced hearing loss in vertebrates [46], and they were exaggerated in flies that lack one copy of the *nervana3* (*nrv3*) gene [45^••^]. *nrv3* was recently shown to encode a Na^+^/K^+^-ATPase β subunit, Nrv3, that, together with the α subunit ATPα, occurs in JONs [46]. Another Na^+^/K^+^-ATPase β subunit, Nrv2, was found in JO supporting cells that enclose the cilia of JONs in a K^+^-rich lymphatic space [47]. Nrv3, ATPα, and Nrv2 all turned out to be essential for JON function, and knockdown of *ATPα* or *Nrv2* led to the accumulation of organelles in the lymphatic space [47]. Apparently, alterations in ion homeostasis render JONs less prone to acoustic overstimulation. Alterations in ion homeostasis were also reported to increase the susceptibility to noise-induced hearing loss in mice [48].

Apart from transcription factors [38^•^,39^•^] and ion pumps [47], various other auditory relevant *Drosophila* proteins were recently defined. Using transcriptome analyses, Senthilan *et al.* [49^••^] identified 274 genes that are enriched in JO, and mutations in 27 of 47 selected genes were found to affect hearing in the fly. Two of the respective proteins, the zinc-finger protein ZMYND10 and the tetratricopeptide repeat domain protein DYX1C1, were recently identified as conserved cilium proteins that are required for axonemal dynein arm assembly and implicated in primary ciliary dyskinesia in humans [50,51]. Other auditory relevant proteins included the two major visual Opsins Rh5 and Rh6, which turned out to be expressed in JONs where they facilitate mechanical amplification and mechanical ion channel gating [49^••^]; the mechanistic basis of this Opsin function, however, is still unclear. Mechanical amplification by JONs was further reported to be independent of Prestin [52], which promotes mechanical amplification in the ears of mammals and birds [24,53]. Instead, mutations in axonemal dynein genes seem to affect this amplification in fly hearing [49^••^], yet unequivocal genetic evidence linking amplification to axonemal dyneins has not been reported yet. Recent studies also suggest that signaling from JO and other chordotonal organs might affect the fly's circadian clock: proteins that also occur in JO were implicated in the entrainment of this clock by mechanical and thermal stimuli [54–56], and a TRP channel that seems implicated in cold sensation, Brivido1, was detected in some JONs [57]. Future studies must show whether JONs are thermo-sensitive and, if so, how thermal stimuli are encoded by these mechanosensory cells.

## Conclusions

Recent studies have documented the functional diversity of *Drosophila* JONs and auditory interneurons in the *Drosophila* brain. The AMMC and the IVLP/WED were supported as primary and secondary auditory centers, and JONs have emerged as cellular paradigms for dissecting mechano-electrical signal transduction, sensory neuron wiring, and sensory cilium function and formation. The stage has been set for using *Drosophila* to study noise-induce hearing impairments, and JONs turned out to use visual opsins for sound detection, and it seems that JONs might be also thermo-sensitive. Obviously, the fly's auditory system is still holding many secrets, leaving much room for discovery.

## References and recommended reading

Papers of particular interest, published within the period of review, have been highlighted as:

• of special interest
•• of outstanding interest

## Conflict of interest statement

Nothing declared.
